# Labyrinthitis With Negative MRI As a Precursor to Rapidly Developing Primary CNS Lymphoma of the Cerebellopontine Angle

**DOI:** 10.1097/ONO.0000000000000020

**Published:** 2022-10-26

**Authors:** Elizabeth S. Longino, Nathan R. Lindquist, Nathan D. Cass, Emily Brignola, Reid C. Thompson, David S. Haynes

**Affiliations:** 1 Department of Otolaryngology-Head and Neck Surgery, Vanderbilt University Medical Center, Nashville, TN; 2 Department of Neurosurgery, Vanderbilt University Medical Center, Nashville, TN.

**Keywords:** Central nervous system lymphoma, Cerebellopontine angle, HIV, Labyrinthitis, Lymphoma

## Abstract

**Background:**

Few case reports have described primary central nervous system lymphoma (PCNSL) presenting as a cerebellopontine angle (CPA) lesion in HIV-positive patients. We describe a rare presentation of rapidly progressing PCNSL of the CPA/internal auditory canal (IAC) as labyrinthitis with initial negative MRI in an HIV-positive patient.

**Case:**

A 58-year-old male with well-controlled HIV presented with sudden left sensorineural hearing loss, tinnitus, and imbalance. Vestibular testing suggested an uncompensated left peripheral vestibular weakness. MRI demonstrated facial and cochleovestibular nerve enhancement within the CPA and IAC. The presumptive diagnosis of labyrinthitis was made. Two months later, he presented to his infectious disease provider with left facial weakness and disequilibrium and was treated for presumed Bell’s palsy. One month later, he presented with left corneal reflex dysfunction, decreased visual acuity, diplopia, and worsening ataxia. Repeat MRI demonstrated a new 3.6 cm lesion of the left CPA/IAC with vasogenic edema. Despite location, the mass lacked the brainstem compression characteristic of other extra-axial CPA masses such as vestibular schwannoma. Flow cytometry and cytology from cerebrospinal fluid was consistent with primary central nervous system large B-cell lymphoma.

**Conclusions:**

We present a unique case of rapidly progressing PCNSL of the CPA/IAC in an HIV-positive patient, presenting initially as labyrinthitis with negative MRI followed by development of multiple cranial neuropathies and 3-month repeat MRI demonstrating a large CPA mass. In HIV-positive patients with a similar initial presentation, PCNSL should considered early in the diagnostic evaluation with close clinical monitoring and a low threshold for repeat imaging.

Hearing loss is a frequent symptom of HIV infection, with possible mechanisms for sensorineural hearing loss (SNHL) including direct viral effects, opportunistic infections or viral reactivation, antiretroviral therapy (ART) ototoxicity, and immunologic disease effects on central auditory pathways (1). Evolving research regarding the pathophysiology of HIV-related hearing loss suggests involvement of the central auditory, vestibular, and cochlear systems with the degree of symptoms possibly related to advanced disease stage and length of infection (2,3). Although sudden SNHL is more common in patients with HIV, the relationship of HIV with acute vestibular dysfunction including vestibular neuritis and labyrinthitis remains less clear (4). These findings in combination with other cranial neuropathies such as facial nerve weakness and/or diplopia may indicate a more nefarious etiology.

A patient presenting with both hearing loss and facial weakness raises suspicion for a mass involving the cerebellopontine angle (CPA) and warrants further imaging evaluation. While both a rare extranodal presentation of lymphoma and an uncommon CPA pathology, primary central nervous system lymphoma (PCNSL) should be considered in the differential diagnosis, particularly in patients with HIV; PCNSL is 3600 times more common in this population and among the top 5 causes of death (5,6). In addition, patients with PCNSL often initially present with neurological symptoms alone and lack characteristic systemic symptoms such as weight loss, fever, and night sweats associated with other malignancies (5). Few case reports have described PCNSL presenting as a CPA lesion, and even fewer describe its clinical presentation in patients with HIV. In this case report, we describe a rare presentation of rapidly progressing PCNSL as labyrinthitis with initial negative MRI in an HIV-positive patient.

## CASE PRESENTATION

**FIG. 1.**
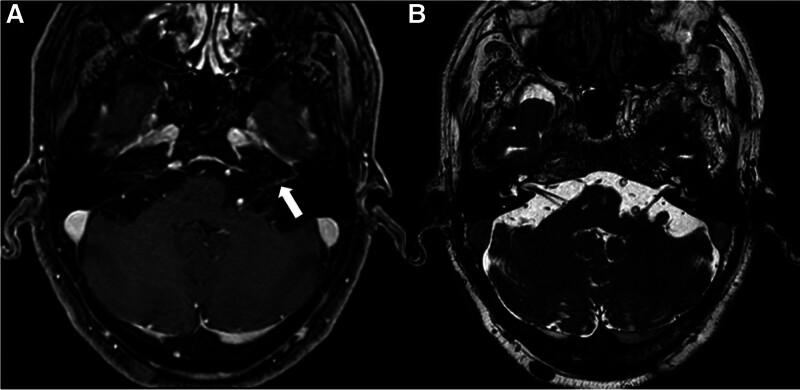
Contrast-enhanced MRI 3 months prior to presentation. Initial MRI with (*A*) contrast-enhanced T1 sequence and (*B*) heavily weighted T2 sequence of the CPA and IAC. Contrast-enhanced T1 sequence demonstrates mild enhancement of the left cochleovestibular nerve region (white arrow). No other appreciable retrocochlear pathology. CPA indicates cerebellopontine angle; IAC, internal auditory canal.

A 58-year-old male with a 14-year history of diabetes mellitus (DM) type 2, alcohol abuse, and well-controlled HIV on ART (CD4+ cell count 963, viral load undetectable) presented to our outpatient otology clinic 3 months after an episode of sudden left SNHL, new-onset tinnitus, and balance issues without overt vertigo or facial nerve weakness. Vestibular testing was consistent with an uncompensated left peripheral vestibular weakness. MRI demonstrated mild enhancement of the facial and cochleovestibular nerves within the CPA and internal auditory canal (IAC) as well as remote right cerebellar infarct (Fig. 1). The presumptive diagnosis of labyrinthitis was made but, given the patient’s comorbid DM and delayed presentation, he elected to forego corticosteroid treatment and was referred to vestibular therapy.

**FIG. 2.**
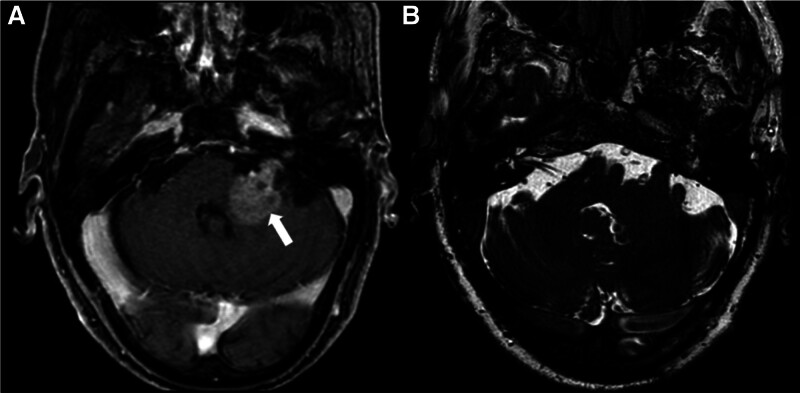
Contrast-enhanced MRI at presentation. Three-month interval MRI with (*A*) contrast-enhanced T1 sequence and (*B*) heavily weighted T2 sequence of the CPA and IAC. Contrast-enhanced T1 sequence demonstrates intra-axial mass within the middle cerebellar peduncle and CPA (white arrow). CPA indicates cerebellopontine angle; IAC, internal auditory canal.

Two months later, the patient presented to his infectious disease doctor with new-onset left-sided facial weakness, and worsening ataxia and disequilibrium. Neurology was consulted and the patient was treated for presumed Bell’s palsy with systemic corticosteroids and antivirals as well as thiamine in the setting of chronic alcohol use. Over the next month, he developed dysfunction of the left corneal blink reflex, decreased visual acuity, diplopia, and worsening ataxia, and presented to the emergency department for further evaluation. Repeat MRI with contrast demonstrated a new 3.6 cm lesion of the left CPA/IAC with surrounding vasogenic edema (Fig. 2), not present on the MRI 3 months prior. Despite its location in the posterior fossa and IAC, the mass did not enlarge at the porus acusticus and was centered within the middle cerebellar peduncle, without the brainstem compression characteristic of extra-axial CPA masses such as large vestibular schwannomas. Further work-up for malignant or metastatic process was obtained; MRI of the spine demonstrated an enhancing cervical spinal cord lesion at C5–C6 suspicious for leptomeningeal disease. Flow cytometry and cytology from cerebrospinal fluid (CSF) demonstrated a CD10-positive monotypic B-cell population. He was diagnosed with primary central nervous system (CNS) large B-cell lymphoma and started on rituximab and high-dose methotrexate.

## DISCUSSION

PCNSL accounts for 0.3%–1.5% of all intracranial malignancies and only 0.7%–2% of lymphomas, and the median age at diagnosis is 65 years. It most commonly presents in the basal nuclei or frontal/parietal lobes. Predisposing factors include advanced age and immunosuppression including HIV. Among HIV-positive patients, PCNSL is the fourth most common cause of death and most common noninfectious cause of death (6).

In HIV patients presenting with clinical signs suspicious for a CPA lesion including unilateral hearing loss, PCNSL should be considered early in the diagnostic evaluation, as disease progression and development of additional signs such as unilateral facial palsy may be rapid, and prompt initiation of chemotherapy and immunotherapy is paramount. The imaging modality of choice is MRI, and lesions related to PCNSL most often demonstrate isointensity or hypointensity on T2 and are contrast-enhancing on T1 sequences (5,7).

As in the case we describe here, the vast majority (90%) of PCNSL is diffuse large B-cell lymphoma. The remaining 10% includes various T-cell lymphomas, low-grade lymphomas or Burkitt’s lymphoma. Patients most often present with a single intracranial mass. Diagnostic evaluation includes lumbar puncture for CSF analysis, possible stereotactic biopsy, or vitreous aspirate in cases with ocular involvement. CSF is sent for flow cytometry, cytology, and immunoglobulin heavy-chain gene rearrangement. Additional imaging, most commonly a positron emission tomography scan, is obtained to confirm CNS as the primary disease. Finally, all patients should be tested for HIV (8).

Overall 5-year survival in PCNSL varies significantly (15%–80%), with increased age, poorer performance status, involvement of deep brain regions, and elevated CSF protein shown to be predictors of poor prognosis (9). While standardized induction treatment has not been established, patients are often started on high-dose intravenous methotrexate with other chemotherapeutics. Radiation therapy is less commonly added to medical therapy, and surgery does not play a role in treatment of PCNSL (8).

Few prior case reports have described PCNSL of the CPA/IAC in HIV patients (6,10,11). Wenzel et al (10) describe PCNSL of the CPA presenting with acute tinnitus and unilateral hearing loss in a patient with undiagnosed HIV, with subsequent rapid development of unilateral facial palsy. Ta and Xu (11) describe PCNSL in a 13-year-old HIV-positive patient presenting initially as multicentric CNS-enhancing lesions suspicious for diffuse demyelinating disease, with a small (6 mm) intracanalicular lesion that was not initially appreciated on imaging until rapid growth was noted on subsequent MRI several months later.

In contrast to these reports, our patient presented with hearing loss and vertigo with an initial MRI demonstrating only peripheral vestibular nerve enhancement, more consistent with a diagnosis of acute labyrinthitis. Furthermore, our patient’s HIV remained well-controlled with normal CD4+ cell count and consistent use of ART. Together, these findings suggest that rapid progression of PCNSL may be observed in patients with well-controlled HIV. A high clinical suspicion and low threshold for repeat imaging is important for patients presenting with progressive cranial neuropathies in the HIV population.

## CONCLUSIONS

We present a unique case of rapidly progressing PCNSL of the CPA/IAC in an HIV-positive patient, presenting initially as labyrinthitis with negative MRI followed by development of multiple cranial neuropathies and 3-month repeat MRI demonstrating a large CPA mass. In HIV-positive patients with a similar initial clinical presentation, regardless of disease control, PCNSL should considered early in the diagnostic evaluation with close clinical monitoring and a low threshold for repeat imaging.

## FUNDING SOURCES

This research did not receive any specific grant from funding agencies in the public, commercial, or not-for-profit sectors.

## CONFLICT OF INTEREST

D.S.H. is a consultant for Advanced Bionics Corp., Cochlear Corp., MED-EL GmbH, Stryker, Synthes, Grace Medical, and Oticon, all which are not relevant to this research. D.S.H. holds the position of Senior Editor for Otology & Neurotology Open and has been recused from reviewing or making decisions for the article. The remaining author discloses no conflicts of interest.

## DATA AVAILABILITY STATEMENT

Data sharing not applicable to this article as no datasets were generated or analyzed during the current study.
