# Labyrinthitis With Negative MRI As a Precursor to Rapidly Developing Primary CNS Lymphoma of the Cerebellopontine Angle: Erratum

**DOI:** 10.1097/ONO.0000000000000032

**Published:** 2023-03-23

In the following article published in *Otology & Neurotology Open,* while informed consent was documented prior to acceptance of each article, this information was erroneously omitted from the final publication.

*Otology & Neurotology Open* 2(4):e020, December 2022.

Labyrinthitis With Negative MRI As a Precursor to Rapidly Developing Primary CNS Lymphoma of the Cerebellopontine Angle

https://journals.lww.com/onojournal/Fulltext/2022/12000/Labyrinthitis_With_Negative_MRI_As_a_Precursor_to.2.aspx
